# Real-World Evidence for Drug Approvals: Moving Toward Greater Regulatory Clarity

**DOI:** 10.1007/s43441-026-01000-3

**Published:** 2026-06-22

**Authors:** Tala H. Fakhouri, Rasika Kalamegham, Nicole Mahoney, Rose Purcell, Mike D’Ambrosio

**Affiliations:** 1 Parexel International, Maryland, USA; 2 Genentech, Washington, DC, USA; 3 Novartis, Rockville, Maryland USA; 4 Takeda, Washington, DC, USA

## Abstract

Despite a decade of investments and regulatory enthusiasm, real-world evidence (RWE) generated from real-world data (RWD) remains underutilized in drug approvals. Beyond the fundamental need for relevant and reliable RWD, three interdependent operational barriers constrain otherwise fit‑for‑use RWD: (1) FDA submission requirements and data standards policies are centered on CDISC and optimized for traditional trials rather than heterogeneous RWD sources; (2) expectations about when patient‑level datasets must be submitted to FDA and how and when FDA may access or inspect source records are unclear; and (3) uncertainty remains regarding the application of 21 CFR Parts 50/56 to non-interventional studies. On one hand, the resulting regulatory ambiguity around these issues may lead sponsors and data providers to expend resources on RWE that is ultimately unfit for regulatory purposes. On the other, potentially suitable RWD may be ignored. In either case, patient access to effective therapies may be delayed. In this perspective, we offer targeted, actionable recommendations in each category to inform FDA’s current efforts to better leverage RWE in regulatory submissions.

## Introduction

The 21st Century Cures Act catalyzed the explicit integration of RWE into regulatory frameworks to accelerate drug development and enhance post-market surveillance [1, 2]. RWE is defined by FDA as clinical evidence about the usage and potential benefits or risks of a medical product derived from analysis of RWD (i.e. data on patient health status and healthcare delivery from electronic health records (EHR), medical claims, registries, etc.) [3]. RWE can complement or be incorporated into evidence generated from randomized controlled trials (RCTs) and may even be the more appropriate mode of evidence generation in certain situations such as for rare diseases or when a clinical trial may be unethical or infeasible.

FDA has issued multiple guidance documents to inform RWD/E adoption [3]. However, RWE is not routinely leveraged by sponsors in their regulatory submissions, and it is unclear how and how often FDA uses this information for regulatory decision-making and approvals. In fact, FDA’s website cites only a limited number of drug or biologics approvals that relied on RWD to generate RWE since 2011 [4]. This perspective explores three interdependent barriers to RWE’s adoption and proposes steps FDA can take to bridge these gaps to facilitate RWE integration into regulatory decisions and ensure suitable RWD is maximized to help bring needed treatments to patients.

## Submission of RWD to FDA: Aligning FDA Data Submission Requirements with RWD Realities

Regulations require sponsors to submit data to FDA using currently supported standards and technical conformance expectations that enable processing, review, and archiving of data. FDA’s data standards policies and technical submission requirements, reflected in its Data Standards Guidance [5] and Data Standards Catalog [6], are largely aligned with CDISC-defined data structures, variable conventions, and traceability expectations developed for traditional, protocol-driven clinical trials. While these standards have supported regulatory review of randomized trials, they are not well suited, without additional transformation, to fully accommodate the structure, provenance, and heterogeneity of RWD sources.

RWD originates from diverse sources (e.g., EHR, medical charts, administrative claims, registries, and data derived from digital health technologies), each with distinct data structures, data-capture workflows, and standardization. Study-specific RWD datasets may include information extracted and processed from structured and unstructured sources, as well as derived variables generated through complex transformations. Converting these heterogeneous data sources into FDA-accepted, analysis-ready formats (i.e. CDISC SDTM and ADaM) often requires extensive manual effort and may obscure provenance, introduce bias, or result in loss of clinically relevant information. As a result, stakeholders across the RWD ecosystem (i.e., sponsors, data generators, data vendors) would benefit from a clearer, shared understanding of regulatory expectations and best practices for handling RWD in regulatory submissions.

One potential approach to addressing these challenges is for FDA to continue exploring flexible, fit-for-purpose data exchange and submission standards for RWD. The agency has already taken initial steps in this direction. In April 2025, FDA’s drug and biologics centers formally opened a public docket to explore Health Level Seven International Fast Healthcare Interoperability Resources (HL7 FHIR) to support submission of structured clinical study data collected from RWD sources [7]. FDA explicitly linked this initiative to broader HHS/Office of the National Coordinator for Health Information Technology’s (ONC) interoperability policy and noted the widespread use of FHIR-based data elements across healthcare systems. The docket solicited public comments on opportunities, challenges, and potential paths forward.

Responses to FDA’s request identified several potential benefits of HL7 FHIR, including improved data interoperability, reduced data processing burden, enhanced linkage across diverse data sources, and greater transparency and auditability of submitted evidence [8]. At the same time, commenters highlighted substantial technical and operational challenges, including the absence of established analysis-ready data structures for FHIR-based RWD, variability in FHIR API implementations across certified health IT developers and health systems, inconsistencies in source data quality and completeness, limited workflow metadata to support provenance assessment, misalignment between clinical and regulatory vocabularies (e.g., SNOMED CT vs. MedDRA), and the need for clear implementation guidance [8].

Additionally, commenters emphasized that FHIR is fundamentally a data exchange standard, optimized for interoperability and upstream acquisition of source data, rather than a standard designed to produce “regulatory-grade”, analysis-ready datasets comparable to CDISC’s SDTM or ADaM. Data exchange standards and analysis data standards serve distinct but complementary purposes [8]. Accordingly, while FHIR represents a critical component of modernizing data flow, it is not currently suitable as a direct submission format for regulatory analysis without additional transformation, validation, and supporting guidance.

The feedback received through FDA’s public docket process makes clear that FHIR’s practical limitations are substantial and will require significant efforts to address. FHIR’s value in this context is most plausibly realized as an upstream data acquisition and exchange layer, reducing the burden of initial data extraction and transfer, rather than as a direct submission format. Even in that more limited role, realizing FHIR’s potential would require developing consensus-based transformation pathways and producing clear FDA guidance on acceptable FHIR-based submission workflows. FDA should, therefore, evaluate FHIR not as a standalone solution but as one component of a broader modernization infrastructure requiring sustained investment. Within that framework, FDA could leverage ongoing standards development efforts, such as CDISC’s work to map FHIR resources and attributes to SDTM domains and variables to support more flexible, fit-for-purpose data exchange approaches that remain aligned with established regulatory review processes. FDA could also explore the possibilities of using AI-driven technologies to harmonize and map heterogeneous RWD sources into various FDA-accepted standards, provided such approaches are transparent, auditable, and commensurate with the regulatory risk and intended use of the data.

In parallel with these longer-term efforts, FDA should consider two short-term approaches. First, FDA could make more systematic use of its existing waiver authority [9] to exempt certain RWD submissions from standard format conversion requirements when appropriate. Second, FDA could invest in modernizing its own review infrastructure to support data in or closer to native RWD formats, shifting the burden of format mismatch away from sponsors.

To further advance these efforts, FDA could consider expanding the data standards catalogue to include HL7 FHIR and establish a collaboration mechanism to work with stakeholders to address the gaps identified through the public docket process. This would align FDA policy with broader federal efforts to modernize the health data infrastructure, including CMS’s interoperability rules requiring FHIR APIs and ONC’s Trusted Exchange Framework and Common Agreement (TEFCA) [10, 11]. Over time, enabling FHIR-based exchange may need to be complemented by harmonized common data frameworks (e.g., OMOP, PCORnet) and clearly defined transformation pathways to support scalable analytics and regulatory-grade evidence generation.

## FDA Review and Inspection of RWD: Aligning Patient-Level Data Access and Source-Record Access with Regulatory Expectations

For drug and biologic approval decisions that rely on RWD, FDA’s position is articulated in the August 2023 RWE Guidance “Considerations for the Use of Real-World Data and Real-World Evidence to Support Regulatory Decision-Making for Drug and Biological Products:” [12]. Specifically, reviewers expect access to patient‑level data sufficient to replicate study analyses, together the traceability needed to support those analyses. The guidance further states that [1] this applies “when required under 21 CFR 314.50 and 601.2”, and that sponsors should align on expectations early; [2] inspectors expect that the source records necessary for verification be available for inspection, as applicable; and [3] FDA does not *currently* accept links to external environments (e.g., cloud hosted by a data vendor, large health system, or registry) in lieu of direct submission or for inspection of such data. The same guidance offers alternate pathways (e.g., Type V drug master file (DMF)) when sponsors cannot submit via traditional channels, but the expectation remains that FDA receives the data themselves, and not a link to an external environment. This may preclude the use of otherwise fit-for-use data from certain data vendors, large health care systems, and/or registries where institutional policy, data agreements, or privacy constraints (e.g., EUGDPR) restrict access to their secure environments.

FDA’s stance on RWD submissions may be shifting however, towards an approach reflected in its December 2025 update to the Agency draft guidance: “Use of Real-World Evidence to Support Regulatory Decision-Making for Medical Devices” [13, 14]. In the press announcement [13], the Agency states that “…it will accept RWE without requiring that identifiable individual patient data collected from real-world data sources always be submitted in a marketing submission” and that “the FDA similarly intends to consider updating its guidance for drugs and biologics”. The lack of detail in the announcement and potential discrepancies with the CDRH guidance text raises new questions about FDA’s expectations for patient-level vs. aggregated data and whether FDA intends to extend CDRH’s “least burdensome provisions” to drugs and biologics, which operate under distinct statutory and regulatory schemes. CDRH’s least burdensome provisions have long applied postmarket controls as a way to reduce premarket data collection expectations. Therefore, it is unclear if the intent is to expand these provisions to drugs and biologics. FDA’s recent guidance for devices provides a prime opportunity to address these and other questions about RWD/E, clarify expectations, explore practical approaches for implementing the device guidance, and explain how the device approach might translate to RWE meant to support drug and biologics approvals.

Access to patient-level data allows FDA reviewers to perform analyses of evidence and establish the reliability of results. Inspections of source data may also be necessary to evaluate study conduct and confirm the quality, integrity and reliability of RWD. A recent publication by FDA staff addressing the use of RWD underscores the limitations of a clinical trials like inspection approach in certain contexts [15]. Because the collection of RWD is not as controlled as for clinical trials, FDA and stakeholders need to think differently about how to access and analyze RWD data to establish the quality and reliability of study results.

The Agency should consider the following recommendations to ensure RWE submitted by sponsors is sufficient for regulatory decisions, and conversely, that fit-for-use RWD that can support regulatory decision-making is not overlooked by sponsors or regulators: 1) Amend the August 2023 RWE Guidance to clarify the circumstances where the submission of aggregated, rather than patient-level data may be acceptable to support regulatory decisions, and where inspections of source data are to be expected. Uncertainty on these issues may limit acceptable uses of RWD by sponsors; 2) Clarify the purpose, focus, and expectations regarding RWD inspections to help data vendors and sponsors meet regulatory expectations in a pragmatic, but scientifically rigorous manner; 3) [ Define in guidance what may be considered source records across different types of RWD (e.g., select information from EHRs, claims, wearables), and confirm that certified copies of select records, and de-identified records are sufficient for source verification during inspection for certain types of RWD [15]; 4) Encourage sponsors to align with FDA as part of the study feasibility plan on the definition of source data, and ways to address any meaningful gaps in availability through the study design or statistical analysis plan. Such an approach would help sponsors evaluate the suitability of RWD and better focus time and resources; 5) Work with stakeholders to align the types and amount of information needed for reviewers to understand how different types of RWD were collected, handled, transformed and establish the reliability of the study results; and 6) Consider how federated data architectures, in which patient-level data remains localized at custodian sites may offer a potential pathway for FDA to access and inspect RWD during the review process. This approach, already demonstrated in FDA’s own Sentinel System and DARWIN EU, addresses privacy barriers, enables RWD analyses and supports traceability. Hybrid models combining federated analytics with selective, certified patient-level submissions (e.g., via secure enclaves or regulator-privileged access) may further support both review and inspections without necessitating direct submission of data to FDA.

Lastly, we acknowledge that when anonymized data are used in research, there is often a network of data use agreements that limit sharing of underlying patient source records to comply with privacy laws. There is a lack of clarity on whether and how various mechanisms for sharing information with FDA would implicate those laws. Absent this clarity, it may be difficult for data vendors to update their data agreements to accommodate mechanisms for FDA review. Since privacy laws and data sharing agreements go beyond the purview of FDA, we need clarification at the level of the Health and Human Services Department to resolve this issue.

## Consent, IRB Oversight, and the Need to Comply with FDA Parts 50/56

Non-interventional RWD studies are not exempt from FDA regulations. Under current FDA regulations, clinical investigations intended to support safety or effectiveness determinations must comply with 21 CFR Parts 50 and 56, which require informed consent and oversight by an institutional review board (IRB). Although August 2023 RWE Guidance [12] acknowledges that certain non-interventional studies are not considered clinical investigations under Part 312 and therefore do not require an IND, the agency emphasizes that “the protection of human subjects under these circumstances is critical,” and sponsors must ensure compliance with Parts 50 and 56.

While appropriate for interventional trials, these requirements create ambiguity for non-interventional studies, which do not fall cleanly into a binary of prospective data collection versus secondary use of existing data. Non-interventional designs span a continuum that includes at least three categories with meaningfully different risk profiles and regulatory considerations: 1) retrospective secondary analyses of deidentified data with no additional data collection; 2) analyses of data generated prospectively through routine clinical care but accessed secondarily for research purposes, with no protocol-driven interaction with patients; and 3) non-interventional studies that incorporate protocol-specified activities such as questionnaires, additional laboratory tests, or imaging beyond what would occur in routine care.

Most RWD sources deployed for secondary use, such as electronic health records or administrative claims, fall within categories one and two. These data are collected under broad initial consent, institutional HIPAA authorizations, or waivers that do not meet the specific informed consent criteria outlined in Part 50, nor do they typically undergo review by IRBs registered with FDA as required by Part 56. The risks to participant rights, safety, and welfare in these categories are substantially lower than in interventional research or in category three designs, where protocol-specified activities introduce additional considerations that may warrant IRB review and informed consent.

FDA should clarify that the protections in 21 CFR Parts 50 and 56 do not necessarily apply to category one studies and should provide explicit expectations for category two and category three designs, including the circumstances under which IRB review, informed consent, or waivers and modifications would be appropriate. Such clarification would preserve ethical integrity while enabling the responsible and scalable use of RWD in regulatory submissions.

## Discussion & Next Steps

The persistent underutilization of RWE in drug approvals stems not solely from limitations related to RWD quality, but also from operational and policy barriers that can be addressed through targeted regulatory measures. By promoting the development and use of flexible, fit-for-purpose data standards, including expanded use of HL7 FHIR and common data models, clarifying expectations for patient-level data access and considering federated or hybrid approaches to data access, and providing explicit guidance on human subject protections for non-interventional studies, FDA can help advance RWE’s potential in regulatory decision making. The recommendations described in this perspective would accelerate evidence generation for rare diseases, underserved populations, and post-market insights while maintaining rigorous standards for relevance, reliability, and ethics. Greater regulatory clarity and innovation in data approaches ultimately enable more efficient, patient-centered drug development and oversight.

## Data Availability

No datasets were generated or analysed during the current study.
